# Non-canonical translation in cancer: significance and therapeutic potential of non-canonical ORFs, m^6^A-modification, and circular RNAs

**DOI:** 10.1038/s41420-024-02185-y

**Published:** 2024-09-27

**Authors:** Xiaoyi Deng, Yanxun V. Yu, Youngnam N. Jin

**Affiliations:** 1 Department of Neurology, Medical Research Institute, Zhongnan Hospital of Wuhan University, Wuhan University, Wuhan, Hubei China; 2 Frontier Science Center for Immunology and Metabolism, Wuhan University, Wuhan, Hubei China

**Keywords:** Cancer metabolism, Targeted therapies, Ribosome

## Abstract

Translation is a decoding process that synthesizes proteins from RNA, typically mRNA. The conventional translation process consists of four stages: initiation, elongation, termination, and ribosome recycling. Precise control over the translation mechanism is crucial, as dysregulation in this process is often linked to human diseases such as cancer. Recent discoveries have unveiled translation mechanisms that extend beyond typical well-characterized components like the m^7^G cap, poly(A)-tail, or translation factors like eIFs. These mechanisms instead utilize atypical elements, such as non-canonical ORF, m^6^A-modification, and circular RNA, as key components for protein synthesis. Collectively, these mechanisms are classified as non-canonical translations. It is increasingly clear that non-canonical translation mechanisms significantly impact the various regulatory pathways of cancer, including proliferation, tumorigenicity, and the behavior of cancer stem cells. This review explores the involvement of a variety of non-canonical translation mechanisms in cancer biology and provides insights into potential therapeutic strategies for cancer treatment.

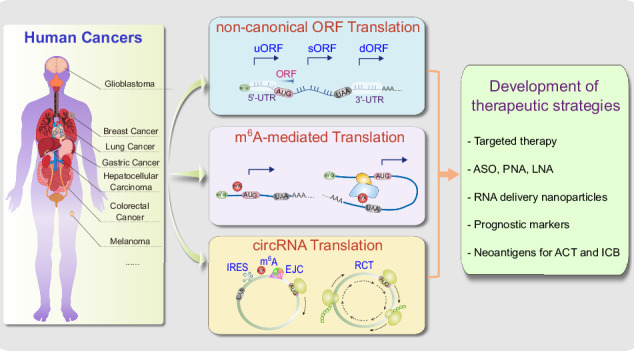

## Facts

- Non-canonical translation mechanisms enhance the translation of oncogenes, growth factors, and anti-apoptotic proteins, contributing to cancer development and progression, yet the intricacies of these processes remain largely unexplored.
- Non-canonical open reading frames (ORFs), including short or small ORFs, upstream ORFs, 3′-UTR downstream ORFs, and long non-coding RNAs, are widespread in human transcripts and exhibit differential regulation in tumor cells.
- N6-methyladenosine (m^6^A) modification of RNA is emerging as a key player in non-canonical translation mechanisms, particularly in cancer biology, by initiating or enhancing the translation of mRNAs as well as non-coding RNAs.
- While circular RNAs are recognized for their role as sponges for miRNAs and proteins in cancer cells, proteins or peptides encoded by circular RNAs have emerged as significant regulators of cancer progression.

## Open questions

- What molecular mechanisms govern non-canonical translation in cancer cells?
- How do proteins produced through non-canonical translation influence the survival and progression of cancer cells?
- How can we specifically target non-canonical translation pathways to inhibit tumor progression?
- How can insights from products translated through non-canonical mechanisms, especially from non-canonical ORFs and circular RNAs, be utilized to develop neoantigens for cancer immunotherapy?

## Introduction

### Canonical translation

**Fig. 1:**
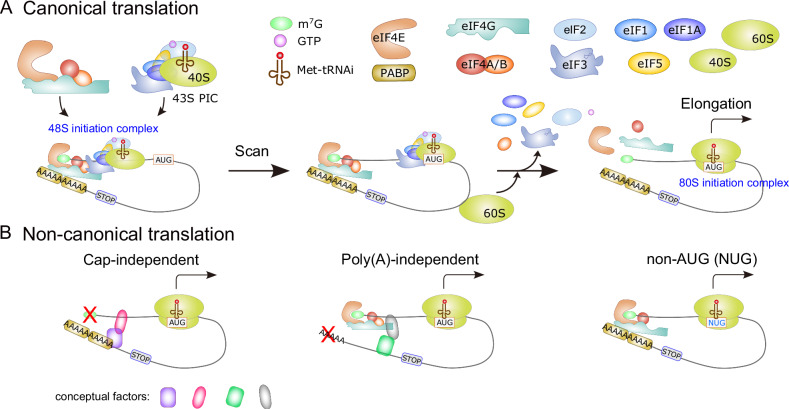
Canonical and non-canonical translation mechanisms. **A** The conventional translation process commences with the formation of an mRNA closed-loop structure, facilitated by multiple interactions involving eIF4E with the mRNA cap and PABP with the poly(A)-tail. Subsequently, eIF4G binds to both eIF4E and eIF4A, forming the eIF4F complex. The eIF4F complex also interacts with PABP via eIF4G. This complex formation assists in recruiting the 43S PIC, comprising the 40S ribosomal subunit, eIF1, eIF1A, eIF3, eIF5, and the eIF2-GTP-Met-tRNAi ternary complex, to the mRNA cap. The 43S PIC then begins scanning from the 5′ to the 3′ direction in an ATP-dependent manner until it recognizes the AUG start codon. GTP hydrolysis then leads to the release of eIFs, allowing the large 60S ribosomal subunit to join and form the 80S ribosome. Translation continues until a stop codon is encountered, at which point the 80S complex is released, and the components are recycled for subsequent translation events. **B** Non-canonical translation mechanisms can occur independently of a cap or poly(A)-tail, and may utilize non-AUG start codons.

Translation of RNA is a fundamental yet complex biological process in which proteins are produced by decoding the codon sequences of RNA molecules, usually mRNAs. Importantly, dysregulated translation is often the cause or a contributing factor to many diseases, including cancer. Translation is carried out in four stages: initiation, elongation, termination and ribosome recycling. Initiation, considered the rate-limiting phase, involves five steps (Fig. 1A) [1, 2]: (1) The 43S preinitiation complex (PIC) forms by combining 40S subunits with eukaryotic initiation factors (eIFs), including eIF1, eIF1A, eIF3, and eIF5, along with the ternary complex of eIF2–GTP-methionyl-tRNA initiator (Met-tRNAi). (2) The eIF4F complex, consisting of the cap-binding protein eIF4E, a scaffold protein eIF4G, and the RNA helicase eIF4A, binds to the 5′ m^7^GpppN (m^7^G) cap (hereafter referred to as the cap) and interacts with poly(A)-binding protein (PABP), associated with the poly(A)-tail, leading to mRNA activation. (3) The 43S PIC is recruited to the activated mRNA, forming the 48S initiation complex (IC). (4) The 48S IC scans the 5′ untranslated region (UTR) until recognition of the start codon AUG, allowing base-pairing with Met-tRNAi. (5) The 60S subunit is integrated in concert with the release of initiation factors including eIF1, eIF2, eIF3, and eIF5, resulting in the assembly of the 80S IC.

During elongation, ribosomes catalyze the formation of peptide bonds between consecutive amino acids carried by tRNAs. Upon bond formation, the ribosome releases the empty tRNA and proceeds along the mRNA in a 5′ to 3′ direction with the aid of GTP hydrolysis and elongation factors (EFs). These steps are reiterated until the ribosome reaches a stop codon. At this point, release factors attach to the ribosome, leading to the release of the fully formed polypeptide chain and termination of translation, enabling the recycling of the ribosome for subsequent rounds of translation.

### Non-canonical translation

Translational mechanisms that bypass the use of the cap, poly(A)-tail, or the standard AUG start codon are classified as non-canonical translations (Fig. 1B) [1, 3]. Emerging research has uncovered alternative pathways for mRNA translation [1, 4, 5]. For example, in previous research, we discovered a process named PAINT (Poly(A)-tail Independent Non-canonical Translation) during early embryogenesis, which operates independently of a poly(A)-tail [6]. We also identified a specific inhibitor of PAINT, known as primordazine, and found that this mechanism is essential for primordial germ cell (PGC) maintenance, as primordazine treatment results in PGC loss [6, 7].

Although non-canonical translation triggered by non-AUG codons [8, 9] or internal ribosome entry sites (IRES) proceeds at a reduced pace compared to canonical translation, it has demonstrated its significance in a variety of biological conditions, including stress responses and cancer cells [5, 9, 10]. Non-canonical translation also plays a significant role in numerous diseases. Notably, many neurological disorders, such as Huntington’s disease, many types of spinocerebellar ataxia, and amyotrophic lateral sclerosis, are linked to non-AUG initiated translation, known as repeat-associated non-AUG (RAN) translation [10–12]. Furthermore, non-canonical translation has significant implications for cancer progression and proliferation, enabling cancer cells to adapt to diverse stress growth conditions [4, 5, 13, 14].

In this article, we review the latest findings on how non-canonical translation pathways regulate tumorigenesis. Additionally, potential therapies targeting these pathways will be briefly discussed, where applicable. Technological approaches for identifying non-canonical translation, reviewed elsewhere [15–17], will not be covered in this paper.

### Non-canonical ORFs

Non-canonical ORFs are widespread in various organisms [18]. However, their potential to translate into proteins has been questioned for a long time, hindering their acceptance within the RNA translation research community [18, 19]. Recent advances in genomic, translational, and proteomic techniques have uncovered additional roles of non-canonical ORFs that extend beyond traditional translational regulation [20–24]. These non-canonical ORFs include short or small ORFs (sORFs or smORFs), upstream ORFs (uORFs), 3′-UTR downstream ORFs (dORFs), and long non-coding RNAs (lncRNAs). Studies show that peptides encoded by uORFs exist in both healthy and tumor cells, influencing cellular functions, metabolism, and immune pathways, and are involved in many diseases, especially cancer [15, 19, 20, 23, 24]. This evidence suggests that non-canonical ORFs possess the capacity to encode proteins or peptides, potentially implicating them in pathological processes. However, the precise translation mechanisms and functions of these products are yet to be fully understood.

### Mechanisms of non-canonical ORF translation

**Fig. 2:**
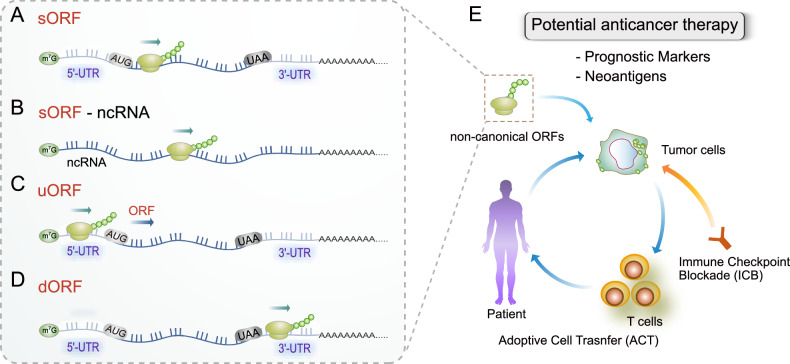
Non-canonical ORFs in various transcript levels. **A** Small ORFs (sORFs) can be found in various positions within RNA, including the 5′-UTR, the 3′-UTR, within the CDS, or interspersed between these regions. **B** sORFs can be also found in non-coding RNA, such as lncRNA. **C** Upstream ORFs (uORFs) are encoded in the 5′-UTR, allowing for translation initiation. **D** Downstream ORFs (dORFs) are located in the 3′-UTR. Translation of dORFs enhances the translation of their respective CDS, differing from uORFs which frequently inhibit the translation of the canonical downstream mORF. **E** Diagram illustrating potential therapeutic approaches for cancer utilizing non-canonical ORF translation. Neoantigens are an invaluable resource for promising cancer treatments through adoptive cell transfer (ACT) or immune checkpoint blockade (ICB).

sORF, a prevalent type of non-canonical ORF consisting of fewer than 150, typically 100, amino acid (aa), encompasses various forms such as sORF, uORF, and dORF. Additionally, lncRNAs are emerging as a form of sORF [15–17]. Initially overlooked, sORFs and their encoded peptides (micropeptides or sORF-encoded peptides) were once dismissed, possibly because codons under 100-aa were considered non-functional. sORFs contain a substantial number of non-AUG initiation codons, with over 50% of sORFs utilizing these non-AUG start codons [8, 10, 20, 25]. They have been found in various RNA positions, such as within a canonical coding sequence (CDS) region as an out-of-frame overlapping ORF (Fig. 2A), in non-coding RNA (ncRNA) transcripts like lncRNAs (Fig. 2B), and in 5′-UTRs (Fig. 2C). Notably, sORFs have been recognized to play crucial roles in various biological processes, including cancer, metabolism, and immunity [8, 15–17, 26–33]. Intriguingly, a significant portion of transcribed enhancers, known as enhancer RNAs, harbor sORFs that can generate microproteins [34].

uORFs are a well-recognized type of non-canonical ORF in the human genome (Fig. 2C) and often contain at least one AUG start codon in about 50% of cases, upstream of the main ORF (mORF) [10], indicating their potential for translatability. Traditionally, uORFs have been regarded as regulatory elements that influence the translation of mORF through mechanisms like leaky scanning and ribosome stalling, rather than serving as CDSs for functional proteins or peptides [15, 18, 35]. Despite this conventional view, a growing body of research has discovered that many uORFs are able to generate functional peptides or proteins [22, 36]. Ribosome analysis suggests that about 30% of mammalian transcripts may contain actively translated uORFs in their 5′-UTR regions [22]. Moreover, non-AUG start codons are frequently used for the translation of many uORFs [8–10, 22, 36]. CRISPR knockout screening has uncovered various micropeptides encoded by uORFs [20]. These micropeptides play roles in diverse biological functions, including cell proliferation [20, 37], stress responses [31], and mitochondrial dynamics [20, 37]. Interestingly, proteins encoded by the uORF often interact with proteins expressed from the downstream canonical mORF, suggesting a potential role over the mORF-encoded protein [20, 37].

dORF, another subtype of sORF, is relatively less common compared to uORF. It is situated in the 3′-UTR downstream of the CDS (Fig. 2D). The translation of dORF enhances the translation of its corresponding CDS [38], in contrast to uORFs, which often suppress the translation of the canonical downstream mORF [35]. Furthermore, substantial translational activity in 3′-UTR regions has been noted in numerous ribosome profiling analyses. Various hypotheses have been suggested to elucidate ribosome binding in the 3′-UTR [15]. One model proposes that ribosome doesn’t stop at a stop codon and continues translation, known as stop codon readthrough, leading to extended translation of the canonical CDS. Two models are suggested to explain how dORF translation can be initiated. The ribosome translation complex temporarily dissociates from the canonical CDS at its stop codon and then reinitiate at the dORF via a presently unknown mechanism. Alternatively, an IRES located in the 3′-UTR may facilitate ribosome recruitment, initiating translation independently of the cap [15, 39]. Although numerous translation products from dORF have been detected through mass spectrometry [25, 40, 41], their functional elucidation remains understudied. A study reported that a peptide derived from dORF of the *ABCB5* gene exhibits immunogenicity in melanoma, acting as a non-canonical human leukocyte antigen (HLA)-binding peptide [40]. Currently, relevant reports are still limited, and confirmations are lacking regarding the translation products of dORFs and their precise roles. Further exploration is necessary to understand the translation mechanism of dORFs.

### Non-canonical ORFs in cancer

Mounting evidence underscores the substantial role of peptides or proteins derived from non-canonical ORFs in contributing to cancer development. This session highlights the latest key findings regarding non-canonical ORFs in cancer research (Table 1).

**Table 1:** Summary of non-canonical translation mechanisms in cancers.

Translation Modes	Cancer Types	RNAs / Mechanisms / Translated products	Functions	Refs.
Non-canonical ORF	Breast cancer	*G029442 (GREP1)* → uORFs → GREP1	Promoting	[23]
		*CASIMO1* → sORF → CASIMO1	Promoting	[44]
		*LINC00908* → sORF → ASRPS	Suppressing	[47]
		*LINC00665* → sORF → CIP2A-BP	Suppressing	[48]
		*KIAA0495* → sORF → SP0495	Suppressing	[54]
		*LINC00992* → cryptic ORF → GT3-INCP	Promoting	[56]
	Pancreatic Neoplasm, Pituitary Adenoma	*CDKN1B* → uORF → 29- or 158-aa peptide	Suppressing	[43]
	Melanoma	*MELOE* → sORF → MELOE-1, MELOE-2	Antigen	[61]
		*TRIT1* → dORF → AS-TRIT	Antigen	[62]
	Various cell lines	eRNAs → sORFs → micropeptides	ND	[34]
	Hepatocellular Carcinoma	*lncRNA ZFAS1* → sORF → ZFAS1	Promoting	[45]
		*LncRNA NCBP2-AS2* → sORF → KRASIM	Suppressing	[46]
		*LINC00998* → sORF → SMIM30	Promoting	[55]
	Esophageal squamous-cell carcinoma	*LINC00278* → sORF → YY1BM	Suppressing	[49]
		*KIAA0495* → sORF → SP0495	Suppressing	[54]
	Colorectal cancer	*LOC90024* → sORF → SRSP	Promoting	[50]
		*LINC00675* → sORF → FORCP	Suppressing	[52]
		*KIAA0495* → sORF → SP0495	Suppressing	[54]
	Head and neck squamous-cell carcinoma	*LncRNA RP11-469H8.6* → sORF → MIAC	Suppressing	[51]
	Medulloblastoma, glioblastoma	*MYCN; MYCC* → uORF → MNOP, MYCNOT; mrtl, MYCHEX1	ND	[57]
	Medulloblastoma	*ASNSD1* → uORF → ASNSD1-uORF/ASDURF	Promoting	[58]
	Nasopharyngeal carcinoma, ovarian cancer, cervical cancer	precursor of miR-34a → sORF → miPEP133	Suppressing	[53]
m^6^A-modifiation	Breast cancer	YTHDF3 → m^6^A → ST6GALNAC5, GJA1, EGFR, VEGFA, SRC	Brain metastasis	[100]
	Prostate cancer	YTHDF1 → m^6^A → PLK1	Promoting	[89]
		YTHDF3 → m^6^A → AR	Survival	[197]
	Ovarian cancer	YTHDF1 → m^6^A → EIF3C	Promoting	[90]
	Hepatocellular carcinoma	YTHDF1 → m^6^A → ATG2A, ATG14	Promoting	[91]
	Colorectal cancer	YTHDF1 → m^6^A → ARHGEF2	Promoting	[92]
	Gastric cancer	YTHDF1 → m^6^A → FZD7	Promoting	[96]
	Glioblastoma	YTHDF3, hnRNP A1 → m^6^A, IRES → cyclin D1, c-myc	Promoting	[101]
circRNA	Bladder cancer	circGprc5a → ? → circGprc5a-peptide	Promoting	[178]
	Colorectal cancer	circSDHAF2 → EJC → SDHAF2 isoforms	Promoting	[165]
		circPLCE1 → IRES → circPLCE1-411	Suppressing	[170]
		circFNDC3B → IRES → circFNDC3B-218aa	Suppressing	[171]
		circMAPK14 → IRES → circMAPK14-175aa	Suppressing	[172]
		circPPP1R12A → ? → circPPP1R12A-73aa	Promoting	[193]
	Hepatocellular carcinoma	circMAP3K4 → m^6^A → circMAP3K4-455aa	Promoting	[157]
		circARHGAP35 → m^6^A → a truncated form of ARHGAP35	Promoting	[175]
		circSTX6 → m^6^A → circSTX6-144aa	Promoting	[176]
		circZKSCAN1 → IRES → circZKSaa	Suppressing	[177]
		circβ-catenin → IRES → β-catenin-370aa	Promoting	[194]
	Gastric cancer	circDIDO1 → IRES, m^6^A → DIDO1-529aa	Suppressing	[174]
		circAXIN1 → IRES → AXIN1-295aa	Promoting	[196]
		circMAPK1 → IRES → MAPK1-109aa	Suppressing	[198]
	Breast cancer	circSEMA4B → IRES → SEMA4B-211aa	Suppressing	[179]
		circHER2 → IRES → HER2-103	Promoting	[192]
		circ-EIF6 → IRES → EIF6-224aa	Promoting	[195]
	Glioblastoma	circMET → m^6^A → MET404	Promoting	[156]
		circEGFR → infinite RCT → rtEGFR	Promoting	[162]
		circPINT → IRES → PINT87aa	Suppressing	[166]
		circSHPRH → IRES → SHPRH-146aa	Suppressing	[167]
		circFBXW7 → IRES → FBXW7-185aa	Suppressing	[168]
		circAKT3 → IRES → AKT3-174aa	Suppressing	[169]
		circSMO → IRES → SMO-193aa	Promoting	[189]
		circE-cadherin → IRES → C-E-Cad	Promoting	[191]
		circHGF → IRES → C-HGF	Promoting	[199]
	Lung adenocarcinoma	circFBXW7 → m^6^A → circFBXW7‑185AA	Suppressing	[173]
	Multiple myeloma	circCHEK1 → IRES → circCHEK1-246aa	Promoting	[200]
	Neuroblastoma	circCUX1 → IRES → p113	Promoting	[201]

*IRES* internal ribosome entry site, *EJC* exon junction complex, *RCT* rolling circle translation, *EMT* epithelial-mesenchymal transition, *ND* not determined.

p27^KIP1^, encoded by the *CDKN1B* gene, functions as a tumor suppressor involved in cell proliferation and differentiation. Reduced levels of p27^KIP1^ are commonly observed in tumor samples, and CDKN1B mutations, though rare, have been identified in several cancers [42]. Notably, a 4-bp deletion in the uORF of *CDKN1B* was identified in a patient with pituitary and pancreatic tumors [43]. This deletion alters the termination codon of the uORF, affecting the length of the uORF-encoded peptide and intercistronic space, ultimately resulting in reduced p27^KIP1^ expression. This highlights the mutation in uORF as a novel mechanism impacting p27^KIP1^ levels, although biological functions of uORF-encoded peptide remain to be elucidated.

Initially classified as a lincRNA, the *CASIMO1* gene was later discovered to encode a 10 kDa transmembrane microprotein, CASIMO1, now officially designated as Small Integral Membrane Protein 22 (SMIM22). The transcript and protein levels of *CASIMO1* are significantly upregulated in hormone receptor-positive (ER^+^ or PR^+^) breast tumors [44]. Knocking it down inhibits cell proliferation, disrupts cytoskeletal organization and cell motility, and triggers G0/G1 cell cycle arrest. Squalene Epoxidase (SQLE) is a rate-limiting enzyme crucial for the cholesterol biosynthesis pathway, converting squalene to 2,3-oxidosqualene. SQLE, recognized for its oncogenic properties and its role in sterol biosynthesis, has been identified as a target of CASIMO1. CASIMO1 enhances SQLE protein levels by directly interacting with and protecting SQLE from degradation, leading to the accumulation of lipid droplets. Conversely, the absence of CASIMO1 leads to a decrease in SQLE protein levels, accompanied by reduced phosphorylation of ERK, a downstream effector of SQLE. CASIMO1 is the first functional microprotein implicated in both carcinogenesis and cellular lipid homeostasis.

ZNFX1 antisense RNA 1 (ZFAS1) is a lncRNA involved in the innate immune response and gene regulation by sponging various microRNAs. A study identified 537 potential sORFs, out of which five were experimentally validated [45]. Analysis of 11 lncRNAs across seven cancer types has revealed that ZFAS1 was notably overexpressed in hepatocellular carcinoma (HCC). Higher levels of ZFAS1 lead to enhanced cancer cell motility, presumably due to an increase in reactive oxygen species. However, it is still unclear whether this tumor-promoting effect is directly attributable to a protein encoded by a sORF within ZFAS1. Another study discovered KRASIM, a 99-aa micropeptide encoded by the lncRNA NCBP2-AS2 [46]. KRASIM inhibits the growth and proliferation of HCC cells by interacting with and reducing KRAS protein levels, consequently decreasing ERK signaling activity.

Triple-negative breast cancer (TNBC) represents a highly aggressive form of breast cancer, associated with unfavorable outcomes and a poor prognosis. LINC00908, identified as a specifically downregulated lncRNA in TNBC, is under the regulation of estrogen receptor α (ERα). LINC00908 harbors a sORF that encodes ASRPS, a 60-aa micropeptide [47]. ASRPS plays a crucial role in inhibiting STAT3 phosphorylation, leading to a reduction in VEGF expression and angiogenesis. Low expression of ASRPS is linked to poor survival in TNBC patients. In mouse models, ASRPS also exhibits anti-tumor properties. ASRPS, produced by LINC00908, presents a promising avenue for targeted therapy in TNBC.

The micropeptide CIP2A-BP, derived from LINC00665, is translationally suppressed by TGF-β in breast cancer [48]. In TNBC, activated SMAD signaling upregulates 4E-BP1, an inhibitory protein for cap-dependent translation that acts by inhibiting elF4E, resulting in the reduced translation of CIP2A-BP. The role of CIP2A-BP is known to disrupt the CIP2A-PP2A interaction, leading to suppression of the PI3K/AKT/NF-κB pathway and inhibition of metastatic factors. Consequently, reduced levels of CIP2A-BP are linked to TNBC metastasis and poorer survival. Introducing CIP2A-BP gene or its micropeptide in a mouse model significantly reduces metastases and improves survival. Thus, CIP2A-BP is a valuable prognostic marker and a promising therapeutic target for TNBC.

The micropeptide YY1BM, originating from the sORF1 of the Y-linked lncRNA LINC00278, is found to be downregulated in male esophageal squamous-cell carcinoma (ESCC) [49]. YY1BM functions by inhibiting the interaction between the transcription factor YY1 and the androgen receptor, resulting in a reduced level of eEF2K. The diminished expression of YY1BM in ESCC leads to a significant increase in eEF2K expression, a factor believed to inhibit apoptosis, thus making ESCC cells more resistant to nutrient deprivation. Additionally, smoking has been shown to reduce YY1BM translation by decreasing the m^6^A-modification of LINC00278.

The lncRNA LOC90024 was discovered to encodes a 130-aa protein called Splicing Regulatory Small Protein (SRSP) [50]. SRSP interacts with splicing regulators, particularly SRSF3, to modulate mRNA splicing. SRSP, not LOC90024 itself, drives colorectal cancer (CRC) progression. SRSP promotes CRC tumorigenesis by enhancing the cancerous long Sp4 isoform. Elevated SRSP levels are linked to aggressive CRC and poor prognosis, making it a potential biomarker and therapeutic target.

A micropeptide called micropeptide inhibiting actin cytoskeleton (MIAC), encoded by RP11-469H8.6, has been identified as a crucial factor in head and neck squamous-cell carcinoma (HNSCC) [51]. Reduced MIAC expression is linked to poorer overall survival rates in HNSCC patients and is significantly associated with the progression of five other distinct tumor types. MIAC’s interaction with AQP2 plays a role in negatively regulating the levels of SEPT2 and ITGB4, leading to the inhibition of the actin cytoskeleton. Consequently, this inhibitory effect suppresses HNSCC tumor growth and metastasis.

The gastrointestinal-specific lncRNA, LINC00675, encodes FORCP (FOXA1-Regulated Conserved Small Protein), a 79-aa micropeptide [52]. Under the regulation of FOXA1, FORCP is typically scarce in most cells but abundant in well-differentiated CRC cells, as FOXA1 is the only transcription factor enriched in these cells. FORCP predominantly localizes to the ER, and its depletion results in reduced apoptosis during ER stress or glucose deprivation. It effectively inhibits proliferation, clonogenicity, and tumorigenesis by acting as a pro-apoptotic factor in response to ER stress.

Derived from pri-miR-34a, the 133-aa microprotein miPEP133 acts as a tumor suppressor by inducing apoptosis, inhibiting cancer cell migration and invasion, and suppressing tumor growth [53]. miPEP133 is downregulated in cancer cell lines and tumors. Lower miPEP133 levels correlate with poorer prognosis in nasopharyngeal carcinoma. Functionally, miPEP133 binds with mitochondrial chaperone HSPA9, inhibiting its function by reducing mitochondrial membrane potential, ATP production, and ultimately inducing apoptosis. Notably, wild-type p53, but not mutant p53, enhances miPEP133 expression. In turn, miPEP133 enhances the transcriptional activity of p53, subsequently increasing miR-34a expression.

Prensner et al. investigated 553 non-canonical ORF candidates and identified 57 that caused viability issues when removed from human cancer cells [23]. Among these candidates, 257 exhibited protein expression upon ectopic introduction, and 401 induced alterations in gene expression. Notably, the ORF G029442, now termed GREP1 (Glycine-Rich Extracellular Protein-1), encodes a secreted protein highly prevalent in breast cancer. Deletion of GREP1 in 263 cancer cell lines resulted in a preferential loss of viability in certain cell lineages, particularly in breast cancer, highlighting its importance for cancer cell survival. The secretome of GREP1-expressing cells featured heightened levels of the oncogenic cytokine GDF15, contributing to GREP1’s growth effect.

The 1p36 region, recognized as a crucial tumor suppressor locus, is frequently subject to deletion in cancer. A CpG methylome analysis unveiled the silenced KIAA0495/*GFOD3P* gene at 1p36.3, revealing its encoding of a small protein named SP0495 [54]. Promoter CpG methylation often hampers the transcription of KIAA0495, resulting in the depletion of SP0495 in various cancers and correlating with poor survival rates among cancer patients. SP0495, through its binding to phosphoinositides, effectively inhibits AKT, mTOR, NF-κB, and Wnt/β-catenin signaling pathways, thereby restraining tumor growth. Additionally, it promotes autophagy by regulating autophagy regulators BECN1 and SQSTM1/p62. This newfound role positions SP0495 as a potential biomarker and a novel methylation-sensitive tumor suppressor in multiple cancers.

LINC00998, initially categorized as a lncRNA, is overexpressed in various cancers. It houses a sORF encoding a micropeptide called SMIM30, located in ER and mitochondrial membranes [55]. Silencing SMIM30 curtails hepatoma cell proliferation and inhibits tumor growth. Introducing a premature stop codon into the sORF abolishes its tumor-promoting effect. Functional assays reveal SMIM30, not LINC00998, controls cytosolic calcium levels, CDK4, cyclin E2, phosphorylated-Rb, and E2F1, driving the G1/S phase transition and cell proliferation. This effect is attenuated by a calcium chelator or SERCA pump agonist, indicating SMIM30’s crucial role in cancer progression.

A recent study that combined ribosome profiling with CRISPR-Cas9 screening has identified several cryptic ORFs encoded by lncRNAs that are crucial for the survival of ERα^+^ breast cancer cells [56]. Among these, LINC00992 has been linked to poor outcomes in luminal breast cancer and is responsible for producing the GATA3-interacting cryptic protein (GT3-INCP). GT3-INCP significantly enhances cell proliferation and tumor growth, an effect that is facilitated by its interaction with GATA3, a key transcription factor involved in the development of luminal epithelial cells. This interaction between GT3-INCP and GATA3 leads to the cooperative regulation of estrogen-responsive and tumor-promoting genes, including MYB and PDZK1.

MYCN or MYCC stands out as one of the most frequently implicated cancer driver genes. The 5′-UTR of MYCN and MYCC mRNAs encompasses uORFs, resulting in MNOP and MYCNOT for MYCN, and mrtl and MYCHEX1 for MYCC [57]. These uORF-encoded proteins are translated to easily detectable levels in tumor cell lines. Upon treatment with JQ1, a bromodomain and extraterminal domain (BET) inhibitor, MYCN, MYCNOT, and mrtl nearly vanish, coinciding with a notable increase in apoptosis levels in pediatric embryonal tumor cell lines that rely on MYC to maintain an undifferentiated phenotype. Although the precise roles of these uORF‐encoded proteins in tumor pathology remain unclear, this study lays the groundwork for the potential involvement of uORF in cancer.

Another study, using ribosome profiling and CRISPR-Cas9 screens on various medulloblastoma samples, revealed the widespread translation of non-canonical ORFs with functions distinct from their primary CDSs [58]. The ASNSD1-uORF protein, also known as ASDURF, derived from a uORF within the *ASNSD1* gene was found to exhibit elevated expression in medulloblastoma. It is indispensable for MYC-driven medulloblastoma cells, but not for non-MYC-driven medulloblastoma or other types of cancer cells. It plays a pivotal role in the survival of medulloblastoma cells through its interaction with the prefoldin-like chaperone complex. This finding highlights the significant role of non-canonical ORF translation in medulloblastoma and underscores the importance of these ORFs and their translation products as promising focuses for developing new therapeutic strategies.

### Prospects for cancer therapy involving non-canonical ORFs

Small peptides arising from non-canonical ORFs, such as CIP2A-BP, SRSP, and miPEP133 [48, 50, 53], serve not only as potential prognostic markers for specific cancer types but also as a significant source of neoantigens, distinct proteins/peptides exclusive to cancer cells and absent in normal tissues (Fig. 2E).

Adoptive cell transfer (ACT) and immune checkpoint blockade (ICB) are highly promising tumor treatments. ACT involves treating cancer patients with their own T cells, which can be naturally occurring or genetically modified, including tumor-infiltrating lymphocytes (TILs), T cells engineered with receptors, or chimeric antigen receptor (CAR) cells. Efforts to develop neoantigen-targeted T cell immunotherapies have gained momentum [59, 60].

Neoantigens originate from tumors through diverse mechanisms, such as DNA mutations, atypical transcriptomic variations, alterations in post-translational modifications, viral ORFs, and non-canonical ORFs [19, 60]. These neoantigens are subsequently presented on the surface of cancer cells through both class-I and II HLA, also known as major histocompatibility (MHC) molecules. Neoantigens exhibit substantial promise as targets for customized cancer immunotherapies.

Recent studies underscore the importance of non-canonical uORF sequences in shaping the immunopeptidome of malignant tissues. For example, meloe has been identified as a polycistronic mRNA generating the melanoma antigens MELOE-1 and MELOE-2 through an IRES mechanism [61]. Furthermore, a peptide originating from the reverse strand in the 3′-UTR of tRNA isopentenyltransferase 1 (TRIT1) serves as an antigen or sensitizes HLA-B57^+^ melanoma cells to lysis by cytotoxic T lymphocyte [62]. Somatic uORF mutations are widespread in various cancers, potentially playing a role in disease onset and progression [63, 64]. Moreover, a recent study revealed numerous functional micropeptides from non-canonical ORFs, some of which are presented by HLA, influencing antigen repertoire and immunogenicity [20]. These studies shed light on a previously neglected dimension of the immune response against cancer.

Using mass spectrometry-based immunopeptidome analysis, 125 distinct HLA uLigands originating from 120 uORFs of 79 genes, such as *ASNSD1*, *ATF5*, *MAPK1*, and *TMEM203*, were discovered. In the pursuit of frequent tumor-associated uORF antigens, these exclusive HLA uLigands were ranked by their occurrence in malignant tissue. This led to the identification of 16 HLA uLigands as tumor-associated uORF neoantigens [65]. Given the analysis’s restriction to about 2000 uORF sequences, the sheer abundance of over 2.4 million AUG- and alternative translation initiation sites-initiated uORFs in the human transcriptome [36] implies that future studies hold the potential to reveal a plethora of additional tumor-specific HLA uLigands.

Targeting tumor-specific neoantigens holds promise for precise tumor eradication while minimizing off-target effects. However, challenges arise from the diverse antigen processing and presentation methods employed by tumors, as well as an incomplete understanding of essential T cell characteristics for clinical effectiveness. Thus, meticulous antigen selection is crucial for ensuring both safety and efficacy. Certain strategies targeting broadly expressed antigens have resulted in ‘off-tumor, on-target’ toxicities, underscoring the need for caution when considering self-antigens co-expressed on vital tissues for clinical testing.

In a recent investigation, personalized neoantigen-HLA capture libraries were created from metastatic melanoma patients, encompassing responders and non-responders to anti-PD-1 immunotherapy [66]. This led to the isolation of neoantigen-specific T cell receptors (neoTCRs). Responders displayed distinct T cell clonotypes recognizing a specific set of mutations, persistently present over time. Conversely, non-responders exhibited lower TCR diversity and sporadic detection of neoantigen-specific T cells, with limited recurrence. T cells from healthy donors, when genetically engineered through non-viral CRISPR-Cas9 gene editing to become neoTCR gene-edited T cells, exhibit specific T cell-mediated cytotoxicity against patient-matched autologous melanoma cells. Predicting immunodominance may guide the selection of antigens for personalized vaccines and therapies. Another recent study provides an effective pipeline to identify tumor immunogenic epitopes/peptides through improved neoantigen prediction [67].

### Translation initiation by m^6^A-modification

#### Overview of m^6^A-modification metabolism in mRNA

**Fig. 3:**
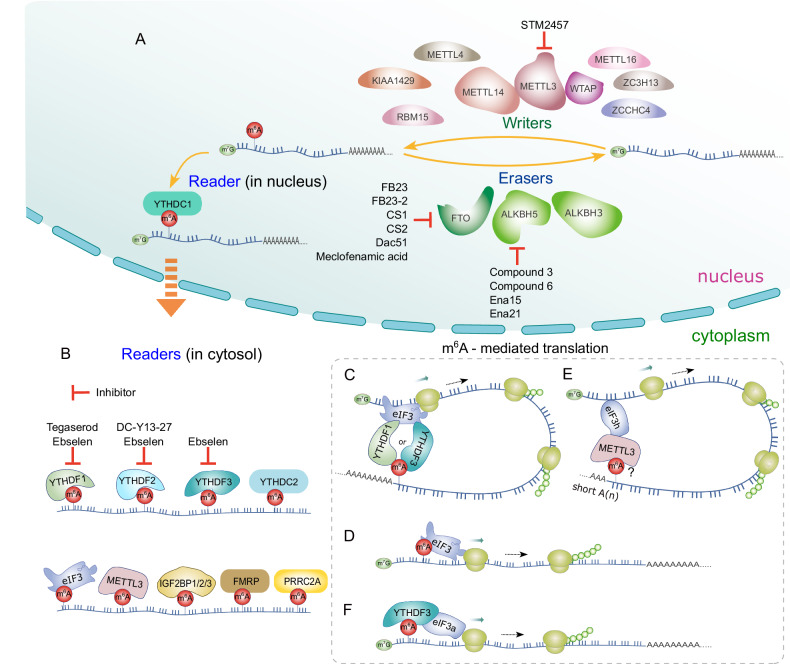
Metabolism of m^6^A methylation and translation mediated by m^6^A-modification. m^6^A-modification can be employed for translation independently of the cap. **A** Overview of the control of m^6^A-modification in the nucleus, involving writers, erasers, and reader, along with their respective inhibitors. **B** Catalog of cytosolic m^6^A readers and their inhibitors. **C** YTHDF1 increases the translation efficiency of m^6^A-modified mRNAs by interacting with eIF3a/b. YTHDF3 collaborates with YTHDF1 and eIF3a to amplify protein synthesis. **D** m^6^A-modification promotes translation by binding with eIF3 in a cap-independent manner. **E** METTL3 associates with eIF3h and binds to mRNA sites near the translation stop codon, facilitating translation even in the absence of a poly(A)-tail. The necessity for m^6^A-modification is not fully understood. **F** YTHDF3 facilitates the binding of eIF3a to the 5′-UTR of mRNAs containing m^6^A residues through a cap-independent translation mechanism.

Being the most prevalent and highly conserved post-transcriptional modification in eukaryotic mRNA, N^6^-methyladenosine (m^6^A) modification plays a crucial role in a wide spectrum of RNA processes, including splicing, nuclear export, RNA degradation and stability, subcellular localization, and translation [68–73]. Disruptions in m^6^A regulation are closely associated with the initiation and progression of cancer by modulating oncogenic signaling pathways, such as Wnt/β-catenin, MAPK, JAK/STAT, PI3K/Akt, and p53 [74], or remodeling the tumor immune microenvironment [75]. Here, our focus will be on exploring the molecular mechanisms of non-canonical translation driven by m^6^A-modification in cancer progression (Fig. 3). Other types of regulations mediated by m^6^A-modification in tumorigenesis, development, differentiation, and other diseases have been reviewed elsewhere [68, 76, 77].

The m^6^A-modification is a reversible and dynamic process, mediated by three categories of proteins: methyltransferases called writers, demethylases called erasers, and binding proteins called readers (Fig. 3A). Writers, exemplified by methyltransferases METTL3 and METTL16, transfer a methyl group from S-adenosylmethionine (SAM) to the N6 position of adenosine residues. Conversely, erasers, such as fat mass and obesity-associated gene (FTO) and α-ketoglutarate-dependent dioxygenase alkB homolog 5 (ALKBH5), remove the methyl group. The m^6^A-modifications are recognized by readers, which include the YTH domain family of proteins and IGF2BP proteins. After the readers bind m^6^A-modified mRNA, the mRNA is subsequently directed towards specific RNA processes, such as RNA decay or translation. Thus, the ultimate trajectory of m^6^A-modified mRNA is predominantly determined by the readers.

In humans, the YTH domain family comprises five members (Fig. 3B): YTHDC1, YTHDC2, YTHDF1, YTHDF2, and YTHDF3. YTHDC1, predominantly located within the nucleus, substantially influences RNA processing. It regulates RNA splicing, ensuring the precise arrangement of exons and introns, and facilitates the export of mature mRNA molecules from the nucleus to the cytoplasm [73, 78]. Conversely, the remaining four YTH proteins primarily reside in the cytoplasm, collectively playing crucial roles in post-transcriptional regulation. Among them, YTHDC2, functioning as an RNA helicase, promotes RNA translation and decay through its helicase activity by unwinding RNA structures for regulatory processes [79–82].

YTHDF1 enhances translation efficiency by interacting with eIF3A/B and increasing ribosomal engagement (Fig. 3C) [83, 84]. In contrast, YTHDF2 induces degradation by recruiting the CCR4-NOT deadenylase complex [85, 86]. YTHDF3 collaborates with YTHDF1 [87, 88] and eIF3a [88] to boost protein synthesis (Fig. 3C) and influences the decay of methylated mRNA mediated by YTHDF2 [88]. Notably, YTHDF2 and YTHDF3 regulate mRNA localization in neuron in a m^6^A-depnedent manner [69]. As a whole, the YTH domain family finely regulates post-transcriptional processes, profoundly impacting gene expression through translation, decay, and localization, ultimately shaping cellular function. The distinct roles and precise localizations of these proteins underscore the meticulous nature of the regulatory mechanisms governing m^6^A-modified mRNA.

Typically, YTH domain family proteins were primarily recognized for their role in modulating canonical translation processes dependent on the cap and poly(A)-tail [79, 83, 87–92]. Intriguingly, YTHDF2, conventionally associated with mRNA degradation [85], emerged as the first member of the YTH domain family to be identified as a facilitator of non-canonical translation, particularly in cap-independent translation initiation during heat shock stress [93]. This discovery has fundamentally altered our understanding of its capabilities. Under heat shock stress, YTHDF2 translocates from its usual cytosolic location to the nucleus. Within the nucleus, YTHDF2 competes directly with FTO to protect the 5′-UTR of stress-induced transcripts from demethylation [93]. This protective mechanism enables mRNAs containing m^6^A in the 5′-UTR, safeguarded by YTHDF2, to be exported and subsequently translated in a cap-independent manner. Subsequent research identified ABCF1 as a pivotal player in m^6^A-mediated translation, under both heat shock stress and physiological conditions, operating in an eIF4F-independent manner [94].

Meyer et al. also demonstrated that a single m^6^A residue within the 5′-UTR can enhance translation independently of the cap or the cap-binding protein eIF4E, revealing an additional function of eIF3 as a reader (Fig. 3D) [95]. Moreover, FTO levels negatively regulate translation during stress conditions, such as heat shock, by demethylating m^6^A in the 5′-UTR [93, 95]. These studies open avenues for exploring the broader role of 5′-UTR m^6^A-modification in cap-independent translation during stress and highlight the importance of investigating the detailed connection between human diseases and cap-independent translation facilitated by 5′-UTR m^6^A.

### Translation of m^6^A-modified mRNAs in cancers

While numerous studies have underscored the direct influence of altered m^6^A RNA metabolism on tumor progression and the acquisition of stem-like characteristics in cancer cells [74, 75, 77, 96, 97], most of these investigations lack an in-depth elucidation of the precise molecular mechanisms governing translational processes via m^6^A-modification in mRNAs. Consequently, distinguishing between canonical and non-canonical translation driven by m^6^A-modification proves to be a challenging task in the majority of studies, although there are some examples where distinct mechanisms are discernible (Table 1).

Lin et al. and Choe et al. made the significant discovery that METTL3, a key writer enzyme, interacts with the translation initiation machinery, particularly eIF3h. This interaction leads to the enhanced translation of a specific subset of mRNAs, facilitated by METTL3 binding to mRNA sites near the translation stop codon (Fig. 3E) [98, 99]. The mechanism underlying this enhancement involves the promotion of mRNA circularization in a cap-dependent manner, even in the absence of a poly(A)-tail [98]. Interestingly, it doesn’t necessitate the m^6^A catalytic activity of METTL3. Rather, the N-terminus region (aa 1-200) of METTL3 alone is sufficient for this augmented mRNA translation [98, 99].

While METTL3 is associated with the translation enhancement of a substantial portion of mRNAs, and m^6^A peaks are predominantly found in proximity to stop codons, METTL3 binding only occurs at about 22% of methylated GGAC sites. Moreover, other readers like YTHDF1 and YTHDF2 are not implicated in this translation mechanism. This suggests the possible existence of additional components that assist in tethering METTL3 to specific target sites [99]. As a result, whether m^6^A-modification near stop codons is an absolute requirement is still subject to further clarification.

In terms of its impact on cancer biology, METTL3 exerts control over the translation of numerous target mRNAs involved in tumor progression and apoptosis. Elevated METTL3 expression has been observed in lung and colon cancers. Depleting METTL3 leads to a significant decrease in the growth, invasion potential, and 3D soft agar colony formation of cancer cells in vitro, as well as a reduction in tumor size in mouse xenografts originating from A549 lung cancer cells. Conversely, elevating METTLL3 levels in human and mouse fibroblast cell lines effectively stimulates cell invasion, a crucial characteristic for tumor advancement. Notably, suppressing METTL3-mediated translation through eIF3h knockdown substantially curtails cell invasion. Furthermore, the capacity of METTL3 to enhance cell invasion and colony formation is nullified by a mutation at A155P, which disrupts the interaction between METTL3 and eIF3h, thereby impeding METTL3-mediated translation [98]. These findings highlight the significant role of METTL3 in advancing tumorigenesis via an mRNA looping mechanism that operates independently of a poly(A)-tail.

A recent study revealed a significant upregulation of YTHDF3 expression in brain metastases originating from breast cancer, as opposed to metastases in other tissues [100]. This heightened expression correlated with a negative impact on survival rates in both breast cancer patients and mouse models, indicating a promotion of brain metastasis. YTHDF3 was found to enhance the translation of its own mRNA, along with m^6^A-transcripts of pivotal genes associated with brain metastasis, including *ST6GALNAC5*, *GJA1*, *EGFR*, *VEGFA*, and *SRC*. Notably, YTHDF3 facilitated the binding of eIF3a to the 5′-UTR of mRNAs containing m^6^A residues, independent of eIF4E, implying a cap-independent translation mechanism (Fig. 3F).

Benavides-Serrato et al. demonstrated that m^6^A-modification enhances the translation of cyclin D1 and c-Myc in GBM cancer cells [101]. Intriguingly, this mechanism functions through m^6^-modification within the IRES elements of their 5′-UTRs, which is recognized by YTHDF3. The heterogeneous nuclear ribonucleoprotein A1 (hnRNP A1) interacts with both YTHDF3 and IRES, leading to enhanced translation and increased resistance of GBM to mTOR inhibitor.

### Prospects for cancer therapy targeting m^6^A-modification

Numerous investigations have underscored the role of m^6^A-modification and its regulatory proteins across diverse human cancers, influencing critical aspects like tumorigenesis, metastasis, resistance, and immunoregulation [75, 102, 103]. Genetic manipulation of writers, erasers, or readers has demonstrated promising results in diverse cancer models [103–105]. Nevertheless, the availability of pharmacological inhibitors remains limited, targeting only a select group of regulators (Fig. 3).

Treatment of STM2457, an inhibitor targeting the methyltransferase activity of METTL3, results in a specific and significant reduction in stem cell populations within acute myeloid leukemia (AML) [106]. This intervention also resulted in a prolonged lifespan for mice without causing discernible toxicity to normal hematopoiesis.

Several inhibitors targeting FTO have been developed, including small-molecule compounds like FB23 and FB23-2. These inhibitors effectively suppress the proliferation of AML cells. Notably, FB23-2 effectively suppresses the growth of human AML cell lines in vitro and inhibits patient-derived primary xenotransplantation (PDX) AML models in vivo [107]. Xiao et al. demonstrated that an antigen-capturing nanoplatform, which concurrently delivers tumor-associated antigens and the FTO inhibitor (FB23-2) into tumor-infiltrating dendritic cells, significantly enhances tumor-specific immune responses both in vivo and in vitro [108].

The recently disclosed FTO inhibitors, CS1 and CS2, exhibit impressive effectiveness in suppressing the proliferation of AML cells in vitro and in PDX models in vivo by targeting FTO’s demethylation activity [109]. Notably, compounds CS1 and CS2 demonstrate anti-tumor potential over ten times higher than that of FB23-2 (>1 µM) in AML cells, with IC50 values in the nanomolar range.

Dac51, an FTO inhibitor, enhances immunotherapy efficacy by countering FTO-mediated immune evasion [110]. It reprograms RNA epitranscriptome, a potential immunotherapy strategy. Dac51 also prevents tumor cells from evading CD8^+^ T cell surveillance by regulating glycolytic metabolism through FTO-mediated m^6^A modifications.

Meclofenamic acid (MA), identified as an FTO-specific inhibitor, exhibits selectivity over ALKBH5 [111]. In a study, MA2-treated mice showed notably reduced tumor size and prolonged survival compared to the control group, underscoring the effectiveness of FTO inhibition in glioblastoma stem cell (GSC) models. This highlights the potential of FTO inhibitors in impeding the progression of GSC-initiated tumors [112].

In contrast to FTO inhibitors, reports on ALKBH5 inhibitors are limited. Nevertheless, Compound 3 and 6, identified through high-throughput virtual screening, demonstrated the ability to inhibit cell proliferation in leukemia cell lines [113]. Similarly, inhibition of ALKBH5 using Ena15 or Ena21, identified through separate screen of small-molecule compounds, resulted in inhibited cell proliferation in glioblastoma multiforme-derived cell lines [114].

Recent advancements in cancer research have unveiled novel YTHDF inhibitors with significant therapeutic potential. Tegaserod, identified through targeted screening of FDA-approved drugs, disrupts the direct binding of YTHDF1 with m^6^A-modified mRNAs, effectively impeding YTHDF1-mediated translation of cyclin E2 [97]. This compound also demonstrates reduced viability of patient-derived AML cells in vitro and extended survival in PDX models.

DC-Y13-27, a potent YTHDF2 inhibitor, enhances tumor response to ionizing radiation (IR) in myeloid cells [115]. By overcoming myeloid-derived suppressor cells (MDSC)-induced immunosuppression, it improves the effectiveness of combined IR and/or anti-PD-L1 therapy, highlighting YTHDF2 as a promising target for radiotherapy and radioimmunotherapy combinations.

Ebselen, a broad-spectrum YTH domain inhibitor, disrupts the interaction between the YTHDF m^6^A domain and its mRNA targets [116]. While its anticancer activity remains untested, it exhibits potential for treating specific cancers like AML, gastric carcinoma, and HCC.

While the potential for cancer treatment through targeting METTL3 and METTL16 has been extensively demonstrated, it is important to note that these genes are essential for cell survival [105, 117]. Consequently, pharmacological inhibition of METTL3, whether by STM2457 or other potential inhibitors, may lead to significant toxicity. Nevertheless, careful adjustment of inhibition levels or drug concentrations could help mitigate this concern.

Interestingly, both METTL3 and METTL16 have been found to participate in translation independently of m^6^A-modification, contributing to tumorigenesis [98, 105], despite their primary roles as m^6^A writers and their predominant action via m^6^A. Given the increasing evidence emphasizing the significance of translation control by METTL3 and METTL16, especially in cancer contexts, developing drugs that selectively target their translation-related activity while preserving their methylation function could offer a promising therapeutic avenue. Additionally, considering the non-canonical translation mechanisms of m^6^A mRNA through interactions with METTL3 or YTHDF1/3 with eIF3, the development of drugs targeting this interaction holds potential for anticancer treatment. Despite the challenges of harnessing m^6^A-modification for cancer therapy, it represents a promising new approach with the potential to revolutionize cancer care in the future.

### Translation of circRNAs

Circular RNAs (circRNAs), ncRNAs generated through back-splicing, were initially discovered in viruses in 1976 and have since been identified in a variety of organisms, tissues, and cell types [118–120]. While the specific functions of many circRNAs still require further study, they are commonly regarded as sponges for microRNAs and proteins [118, 119, 121]. Unlike typical mRNA, circRNAs lack both a cap and a poly(A)-tail, classifying them as ncRNA molecules. The potential for circRNA translation is still under discussion and requires thorough exploration [122, 123], yet mounting evidence indicates that certain circRNAs contain ORFs and can encode peptides/proteins via cap-independent translation mechanisms [14, 118, 120]. Historically, proteins encoded by ORFs shorter than 100-aa were excluded from protein databases, but circRNAs often contain such short ORFs. Recent advancements in bioinformatics, translational research, and proteomics have revealed numerous peptides/proteins encoded by circRNAs [124–126]. The involvement of circRNA-produced peptides/proteins has been noted in several disease contexts, including Alzheimer’s disease [127, 128], cardiovascular diseases [129, 130], pulmonary fibrosis [131], and various types of cancer [120].

In this section, we will delve into the molecular mechanisms, key recent discoveries, and therapeutic implications of circRNA-driven translation in cancer progression. The biogenesis, detection, biomarkers, and other regulatory functions of circRNAs, including their role as microRNA sponges in cancer, are covered in other reviews [119, 121].

### Mechanisms of circRNA translation

**Fig. 4:**
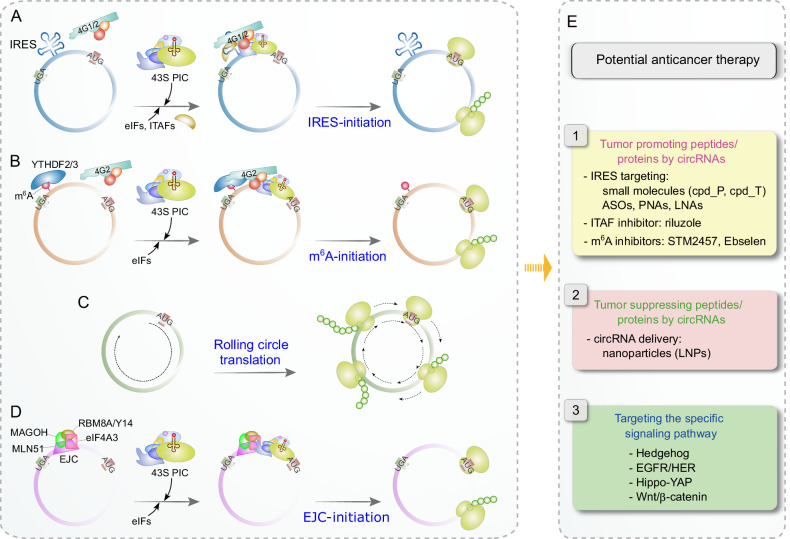
CircRNA translation and potential anticancer therapeutic strategies. The translation of circRNAs occurs independently of a cap and poly(A)-tail. **A** IRES-mediated non-canonical translation is a prevalent mechanism in circRNA, circumventing the need for a cap and several eIFs. **B** YTHDF2 or YTHDF3 recognizes m^6^A-modified residues, recruiting initiation factors such as eIF4G2 and 43S PIC, which then initiates translation. **C** In cases where a circRNA lacks a stop codon and possesses a nucleotide count that is a multiple of three, it allows for limitless translation, potentially leading to the production of polymeric structural proteins through infinite rolling-circle translation (RCT). **D** EJC deposited during back-splicing promotes the initiation of circRNA translation through the interaction between eIF4A3 and eIF3g. **E** The diagram outlines potential therapeutic strategies for anticancer interventions that utilize circRNA translation.

In contrast to traditional translation mechanisms, uncapped circRNAs initiate translation through two non-canonical translation mechanisms: IRES [132] and MIRES (m^6^A-induced ribosome engagement site [95]) (Fig. 4) [14, 120].

IRES, a well-studied cis-element in cap-independent translation, was initially identified in the poliovirus [133, 134] and later discovered in both viral (vIRES) and cellular (cIRES) genes [1, 2, 135, 136]. Typically located in the 5′-UTR and rarely in CDS regions [137], IRES elements enable mRNAs to directly recruit the 40S ribosomal subunit for translation initiation. IRES translation requires only a subset of eIFs and operates independently of the cap [1, 2]. IRES interacts with eIF4G1 (also called eIF4GI or p220)/eIF4G2 (also called DAP5 or p97) [138–142], or the eIF3 complex [142–144], leading to the recruitment of 40S subunits and the subsequent formation of 43S PIC to initiate translation (Fig. 4A).

Cap-independent translation enhancers (CITEs) are structural RNA elements with diverse structures that facilitate translation initiation by binding to specific eIFs such as eIF4G1, eIF4G2/DAP5, eIF4G3, eIF4E, and eIF3, as well as 18S rRNA [145–147]. CITEs are similar to IRESs in that both mechanisms enable mRNA translation without requiring a cap structure and eIF4E. However, CITEs require a free or exposed 5′ end and employ ribosome scanning to locate the start codon. The first CITE, known as the translation enhancer domain (TED), was identified in *satellite Tobacco necrosis virus* (sTNV) [148]. While numerous studies have reported cap-independent or IRES-mediated translation in mammalian cells, only a few have provided convincing or direct evidence of CITEs functioning via eIF4G1 or eIF4G2 [138, 149, 150]. Although it is highly plausible that cells under stress, such as cancer cells under hypoxia, may employ cap-independent translation mechanisms like CITEs, there is currently no report directly linking CITE regulation to cancer or other pathological conditions. Additionally, CITEs can be located in both the 5′- and 3′-UTRs of mRNA, with a predominance in the 3′-UTR. However, in mammalian cells, only a few 5′-UTRs, such as those from HIF1A, TP53, and APAF1, have been identified as CITEs, while no 3′-UTR CITEs have been discovered. This discrepancy may be due to the overestimation of IRESs and insufficient experimental validation. Therefore, identifying cellular CITEs and understanding their involvement in physiological processes are important directions for future research, and our review will not include details of CITEs.

Given that circRNAs lack both a cap and a poly(A)-tail, they exclusively undergo cap/poly(A)-independent translation [1, 120]. Therefore, IRESs play a crucial role in translation initiation for circRNAs. Chen et al. discovered over 17,000 native or synthetic sequences functioning as IRESs for circRNAs through a split-eGFP reporter system containing an IRES oligo library. They demonstrated that circRNA translation via IRES involves base-pairing between the IRES and the active regions of the 18S rRNA and a structured RNA element within the IRES [132]. With the increasing recognition of the biological significance of circRNA translation, a recent study introduced DeepCIP, a deep learning approach, to predict IRES elements in circRNAs by combining two deep neural network modules, Sentence-State LSTM (S-LSTM) and Graph Convolutional Networks (GCN) [151]. Although requiring further improvement, this method will enhance the understanding of translation mechanisms of circRNAs.

IRES transacting factors (ITAFs) play important roles in modulating IRES-driven translation by stabilizing IRES structures or inducting necessary conformation changes, thereby promoting the recruitment of ribosomal subunits. While most identified ITAFs are associated with linear mRNA, their roles and mechanisms in IRES-mediated translation have been discussed elsewhere [152]. However, some ITAFs have been identified from circRNA translation. PABPC1/4 and hnRNP U facilitate translation by recognizing IRES-like elements, especially AU-rich sequences, in circRNA [125]. Although IRES-mediated non-canonical translation is a major mechanism in circRNA translation, the comprehensive regulatory mechanisms by IRES and specific ITAFs involved still demand further exploration.

m^6^A methylation is notably more prevalent in circRNAs than in mRNAs [153, 154], with METTL3 and YTHDC1 positively influencing the biogenesis of circRNAs through m^6^A-modification [154, 155]. This m^6^A-modification plays a crucial role in enabling cap-independent translation by acting as a MIRES. Within the 5′-UTR, m^6^A can interact with YTHDF3 or eIF3, facilitating the recruitment of the 43S PIC independently of the cap, thereby initiating translation (Fig. 3D, F) [95, 100]. Therefore, it is plausible that RNA elements modified with m^6^A may exhibit similar MIRES-initiated translation activity in circRNA. Supporting this hypothesis, studies have shown that m^6^A sites in circRNAs can be recognized by YTHDF3, which, upon binding to eIF4G2, recruits the 40S subunit for translation initiation (Fig. 4B) [153, 155]. Remarkably, a single m^6^A-modification in circRNA is sufficient to initiate translation with the cooperation of eIF4G2 and YTHDF3 [153]. The removal of m^6^A by the demethylase FTO decreases circRNA translation, whereas co-expression with the m^6^A methyltransferases METTL3/14 leads to an increase. Furthermore, a reduction in YTHDF3 levels results in decreased circRNA translation [153]. A recent study also discovered that YTHDF2, rather than YTHDF3, facilitates m^6^A-dependent translation of circMET in GSCs [156]. Moreover, another investigation revealed that circMAP3K4 produces the circMAP3K4-455aa protein through m^6^A-modification, which is recognized by IGF2BP1, thereby facilitating the progression of HCC [157]. This finding provides a new insight into m^6^A-mediated translation in circRNA, although the exact function of IGF2BP1 in circRNA translation remains incompletely understood.

Rolling circle translation (RCT) represents a distinctive translation process for circular RNAs that do not contain a stop codon, enabling continuous translation due to an uninterrupted ORF (Fig. 4C). This mode of translation, reminiscent of a polymerase reaction, occurs when a circRNA is devoid of a stop codon and features a nucleotide sequence that is a multiple of three, creating an infinite ORF (iORF). Theoretically, this allows for endless translation, ultimately leading to the production of polymeric structural proteins as demonstrated in *Escherichia coli* and mammalian cells in live-cell or cell-free systems [158–161]. Similarly, a recent study showed that circEGFR employs infinite RCT to encode a rolling-translated EGFR (rtEGFR), resulting in the formation of a novel polymeric protein complex. The programmed -1 ribosomal frameshifting (-1PRF) mechanism, which generates an out-of-frame stop codon, was found to facilitate translation termination [162]. Fan et al. reported that approximately 67% of endogenous circRNAs have the capacity to encode proteins exceeding 20-aa, overlapping with their corresponding genes. An additional 27% of circRNAs also exhibit translatability, leaving only 7% of circRNAs without any identifiable ORF. Intriguingly, analysis from public datasets of mass spectrometry suggests that around 50% of translatable circRNAs can generate protein concatemers through the RCT mechanism [125]. In addition, a recent study predicted that circASPH splice variants lacking a stop codon may undergo infinite RCT, producing large proteins, though experimental validation is required [163]. This non-classical RCT translation mechanism introduces a potential third pathway in circRNA, distinct from IRES- and m^6^A-mediated translations. However, the identification of RCT products of endogenous circRNAs is limited, and further exploration is needed to unravel their biological functions and underlying mechanisms.

Additionally, two recent studies have demonstrated that the exon-junction complex (EJC), which is deposited during back-splicing, facilitates ribosome recruitment to circRNA through the interaction between eIF4A3 and eIF3g, thereby enhancing translation initiation (Fig. 4D) [164, 165]. However, further research is needed to thoroughly address the many remaining questions. How does the EJC selectively activate a subset of circRNAs, given that almost all circRNAs are produced by back-splicing? Does the EJC work in conjunction with IRES or m^6^A-driven translation mechanisms? To what extent does the EJC contribute to circRNA translation? Does the EJC remain attached following the initial round of translation? Importantly, do circRNA translations driven by the EJC have significant roles in cellular functions or disease progression?

### Translation of circRNAs in cancer

In this section, we will highlight significant discoveries in circRNA translation, as a growing body of research emphasizes its clinical importance in many cancers (Table 1).

GBM, a grade IV astrocytoma, is an aggressive brain cancer with a survival rate of less than 10% beyond five years after diagnosis. PINT87aa, encoded by circRNA LINC-PINT, restrains GBM cell proliferation in vitro and in vivo [166]. It directly interacts with the polymerase-associated factor complex (PAF1c) in the nucleus, impeding the transcriptional elongation of multiple oncogenes, including c-Myc. Both PINT87aa and LINC-PINT are reduced in GBM compared to normal tissue levels, suggesting the involvement of functional peptides encoded by circRNA in GBM tumorigenesis.

The ORF in circ-SHPRH produces SHPRH-146aa, a functional 17 kDa protein via IRES. Both circ-SHPRH and SHPRH-146aa exhibit reduced expression in GBM [167]. SHPRH-146aa functions as a GBM tumor suppressor by protecting SHPRH, a known tumor suppressor, from ubiquitination. This protection leads to the inhibition of cell proliferation and tumorigenicity in vitro and in vivo, as seen in experiments with U251 and U373 GBM cell lines. Both SHPRH-146aa and SHPRH expression levels are positively correlated with a better prognosis for patient with GBM.

Circ-FBXW7 carries an IRES-driven transjunctional ORF that encodes FBXW7-185aa [168]. Elevated levels of FBXW7-185aa inhibit GBM cell proliferation, while decreased levels promote malignancy in vitro and in vivo. FBXW7-185aa reduces c-Myc stability by counteracting the activity of the de-ubiquitinating enzyme USP28, which stabilizes c-Myc through de-ubiquitination. In GBM clinical samples, both circ-FBXW7 and FBXW7-185aa levels are reduced. Importantly, circ-FBXW7 shows a positive correlation with overall patient survival.

Another circRNA, circ-AKT3, produces AKT3-174aa, which competes with AKT by interacting with PDK1. This competition results in decreased Akt-Thr308 phosphorylation and PI3K/AKT signaling [169]. Consequently, it suppresses GBM cell proliferation, radiation resistance, and tumorigenicity. On the contrary, when circ-AKT3 is downregulated, it promotes cell malignancy.

CRC stands as the third most prevalent cancer and the second highest contributor to cancer-related fatalities worldwide. One notable circRNA, circPLCE1, produces a 411-aa protein circPLCE1-411, driving the dissociation of RPS3 from the HSP90α/RPS3 complex [170]. This triggers RPS3 degradation, a pivotal regulator of NF-κB. As a result, circPLCE1-411 inhibits NF-κB nuclear translocation in CRC cells, suppressing both proliferation and metastasis. These findings were confirmed across multiple CRC models.

Another circRNA, circFNDC3B, exhibits reduced expression in both CRC cell lines and patient tissues [171]. This circRNA produces circFNDC3B-218aa, functioning as a tumor suppressor by inhibiting CRC proliferation, invasion, and migration. The tumor-inhibitory effect of circFNDC3B-218aa is mediated through the suppression of Snail expression. This leads to the upregulation of FBP1, a recognized tumor suppressor, further enhancing its tumor-suppressive impact in CRC.

Similarly, the expression of circMAPK14 is significantly decreased in CRC cells and tissues. It is primarily localized in the cytoplasm and encodes a 175aa peptide, circMAPK14-175aa [172]. This peptide acts as a tumor suppressor by reducing the nuclear translocation of MAPK14 through competitive binding to MKK6, thereby facilitating the ubiquitin-mediated degradation of FOXC1. Additionally, there is a positive feedback loop in CRC, where reduced circMAPK14-175aa expression leads to elevated FOXC1 expression. This, in turn, suppresses U2AF2 transcription, leading to decreased circMAPK14 biogenesis.

Lung cancer has the highest death rate among all cancers globally. In the intricate landscape of lung adenocarcinoma (LUAD), the alternative Wnt pathway is a pivotal player by sustaining stem cell renewal and fostering resistance. Notably, the diminished expression of circ-FBXW7 is implicated in resistance to Osimertinib, a third-generation EGFR inhibitor and targeted therapy for lung cancers, including LUAD [173]. Circ-FBXW7 inhibits stem cell renewal, enhancing the response to Osimertinib. It generates circ-FBXW7‑185AA, also known as FBXW7-185aa [170], in an m^6^A and YTHDF3-dependent manner. This peptide interacts with β‑catenin, suppressing Wnt signaling and leading to increased Let-7d miRNA expression. Let-7d, in turn, reduces YTHDF3 levels, establishing a negative feedback loop. This study reveals the mechanism behind LUAD stem cell resistance to Osimertinib, providing insights into potential therapeutic strategies.

In gastric cancer, or stomach cancer, circDIDO1 plays a crucial role. Downregulated in gastric cancer tissues, circDIDO1 hinders gastric cancer cell proliferation, migration, and invasion [174]. Its overexpression leads to reduced tumor growth and metastasis. CircDIDO1 contains IRES, ORF, and m^6^A-modification, generating a protein called DIDO1-529aa. This protein interacts with PARP1, inhibiting its activity, and targets PRDX2 for degradation, deactivating downstream pathways. This discovery underscores the tumor-suppressive role of DIDO1-529aa in gastric cancer.

HCC or liver cancer is the third most common cause of cancer-related deaths worldwide. circARHGAP35, derived from ARHGAP35 mRNA through back-splicing facilitated by HNRNPL, possesses a 3867 nucleotide ORF initiated by m^6^A-modification. The resulting circARHGAP35 protein, a truncated form of ARHGAP35, activates oncogenes by binding to TFII-I via FF domains within the nucleus, correlating with an unfavorable prognosis in HCC patients [175].

CircSTX6, along with its 144aa peptide circSTX6-144aa, is highly expressed in HCC tissues and serves as an independent risk factor for patient survival [176]. METTL14 regulates circSTX6 expression through an m^6^A-dependent mechanism. Functionally, circSTX6 promotes HCC proliferation, tumorigenicity, and metastasis. Mechanistically, it acts as a sponge for HNRNPD, promoting HNRNPD-mediated ATF3 mRNA decay. Additionally, circSTX6-144aa independently drives HCC progression. This highlights their potential as biomarkers and therapeutic targets in HCC.

A tumor-suppressive circRNA, circZKSCAN1, is significantly underexpressed in HCC samples and encodes a 206-aa protein circZKSaa, which is predominantly located in the cytoplasm and can be secreted [177]. circZKSaa effectively inhibits the growth of HCC cells in vitro and in vivo by promoting the degradation of mTOR through interactions with both mTOR and FBXW7.

In bladder cancer, circGprc5a is upregulated in tumors and cancer stem cells (CSCs), with its overexpression boosting the CSC ratio via the circGprc5a-peptide encoded by circGprc5a [178]. The study reveals a critical interaction between circGprc5a-peptide and Gprc5a, a protein crucial for bladder CSC maintenance. Conversely, mutant circGprc5a or overexpression of circGprc5a in the absence of Gprc5a does not affect CSC ratios. This underscores the therapeutic potential for bladder cancer by targeting circGprc5a-peptide, Gprc5a, or their interaction.

In breast cancer, circSEMA4B was notably decreased in tumor tissues and cell lines and acts as a tumor suppressor through two distinct mechanisms [179]. First, circSEMA4B sponges miR-330-3p, resulting in the upregulation of the tumor suppressor PDCD4 and subsequent inhibition of AKT signaling. Second, SEMA4B-211aa, encoded by SEMA4B, indirectly interacts with free p85, a regulatory subunit of PI3K, outcompeting the catalytic subunit p110. This interaction suppresses PI3K activity and the downstream AKT signaling. This study sheds light on the modulation of the PI3K/AKT signaling pathway as a strategy against breast cancer progression.

### Potential in cancer therapy targeting circRNA translation

The translation of circRNAs is crucial in cancer progression, yet there are currently no specific inhibitors or modulators to regulate circRNAs processes like IRES or MIRES. Inhibiting the translational synthesis of tumor-promoting peptides/proteins encoded by circRNAs could effectively prevent cancer (Fig. 4E). Small-molecule inhibitors have shown the capability to hinder the translation of IRES-containing transcripts such as IGF1R and c-Myc, without impeding global cap-dependent translation [180, 181]. However, the vast diversity of IRES sequences in circRNAs, along with vIRES and cIRES, may render it challenging to develop small molecules targeting each specific IRES. Alternatively, antisense oligonucleotides (ASOs) or peptide nucleic acids (PNAs) can precisely target individual IRES sequences [181, 182]. Targeting the interaction between IRESs and ITAFs holds promise as a potential avenue for developing specific small molecule inhibitors such as riluzole [181, 183]. Nevertheless, these IRES inhibitors have not been tested for their ability to inhibit circRNA translation. Additionally, several drugs targeting m^6^A-modification are available, as described in section “Prospects for cancer therapy targeting m^6^A-modification”. It may be worthwhile to investigate whether pharmacological inhibition of IRES or m^6^A-modification could effectively prevent circRNA translation, and thereby suppress tumor progression.

On the other hand, enhancing the expression of tumor-suppressing peptides/proteins or circRNAs through increased circRNA expression is a conceivable means to more effectively combat cancers. Lipid nanoparticles (LNPs), enclosed spherical lipid vesicles, are widely used in the preclinical or clinical setting as carriers for drugs, including circRNA [184–186]. For instance, circFoxo3 was delivered through plasmids conjugated with gold nanoparticles to induce tumor apoptosis [187]. Additionally, overexpression of circ-1073 inhibited breast cancer cell proliferation and induced apoptosis. The growth of xenograft tumors was suppressed by intratumoral injection of nanoparticles containing circ-1073 [188].

Targeting critical signaling pathways influenced by oncogenic peptides or proteins produced by circRNAs offers substantial promise for cancer therapy. Here, we highlight examples of circRNA-encoded peptides/proteins that act as oncogenic molecules and explore their therapeutic implications. Furthermore, the translation products from circRNAs may serve as neoantigens for cancer immunotherapy, as detailed in section “Prospects for cancer therapy involving non-canonical ORFs”. For more information on neoantigens, please refer to that section.

Dysregulated activation of the Hedgehog pathway has been associated with various cancers, including medulloblastoma, gastric cancer, HCC, pancreatic cancer, and others. Circ-SMO generates SMO-193aa, essential for initiating Hedgehog signaling in GBM [189]. SMO-193aa promotes GBM progression. Diminished Hedgehog signaling in GSCs hampers self-renewal, in vitro proliferation, and in vivo tumorigenicity. Targeting circ-SMO and/or SMO-193aa could be a promising therapeutic approach for GBM treatment.

EGFR and other members of the ErbB/HER family are closely linked proteins essential for the regulation of cell growth and division. Alterations such as mutations or increased expression of these receptors can drive cancer advancement. Cancer types associated with EGFR/HER modifications include non-small cell lung cancer, GBM, breast cancer, and various others [190]. Circ-E-Cad RNA undergoes successive rounds of ORF translation, facilitated by the absence of a stop codon in the initial round read. This process leads to the generation of a novel secreted E-cadherin protein variant, C-E-Cad [191]. C-E-Cad binds to the EGFR CR2 domain via a 14-aa carboxyl terminal, activating EGFR independently of EGF and thus contributing to the maintenance of the tumorigenicity of GSCs. C-E-Cad is overexpressed in GBM, further enhancing the tumorigenicity of GSCs.

Another circRNA, circ-EGFR, utilizes an iORF to generate rtEGFR through infinite RCT [162]. rtEGFR interacts with EGFR, maintaining its membrane localization and reducing endocytosis and degradation. Depletion of rtEGFR in brain tumor initiation cells reduces tumorigenicity and enhances anti-GBM effects.

Circ-HER2, found in 30% of TNBC cases, produces a 103-aa protein, HER2-103 [192]. TNBC patients with circ-HER2/HER2-103-positive status often exhibit poorer prognoses. Suppressing circ-HER2 hinders TNBC cell behaviors, underscoring its pivotal role in tumorigenesis. Mechanistically, HER2-103 drives EGFR/HER3 dimerization, sustaining AKT activation and promoting malignancy. HER2-103 shares sequences with the HER2 CR1 domain, which is a target of pertuzumab. Pertuzumab significantly reduces in vivo tumorigenicity of circ-HER2/HER2-103-positive TNBC cells. These discoveries present a potential approach for cancer treatment by targeting EGFR/HER.

The Hippo-YAP pathway, which governs cell growth, proliferation, and organ proportions, is implicated in various types of cancer, including HCC, breast cancer, CRC, and others. In colon cancer, circPPP1R12A is specifically upregulated. It encodes the functional peptide circPPP1R12A-73aa, which enhances colon cancer growth and metastasis by activating the Hippo-YAP signaling pathway [193]. Patients with elevated levels of circPPP1R12A exhibit markedly reduced overall survival. Importantly, the YAP-specific inhibitor, Peptide 17, substantially attenuates the cancer-promoting impact of circPPP1R12A-73aa on colon cancer cells, suggesting that Hippo-YAP inhibition could serve as a therapeutic strategy for this type of cancer.

The Wnt/β-catenin pathway is pivotal in regulating cell growth and differentiation. Abnormal activation of this pathway is associated with various cancers, including colorectal, breast, liver, and lung cancers. In liver cancer, circβ-catenin is expressed at high levels, and silencing circβ-catenin significantly suppresses malignant characteristics both in vitro and in vivo [194]. A novel 370aa β-catenin variant, β-catenin-370aa, encoded by circβ-catenin, stabilizes full-length β-catenin by counteracting its phosphorylation and degradation by GSK3β. As a result, the Wnt pathway is activated, promoting tumor growth in liver cancer.

In TNBC, circ-EIF6 is correlated with poor prognosis due to its enhancement of cancer cell growth and metastasis [195]. An IRES within circ-EIF6 facilitates the expression of EIF6-224aa, which interacts with MYH9, a known oncogene. This interaction stabilizes MYH9 and activates the Wnt pathway, contributing to the oncogenic effects of circ-EIF6 in TNBC.

Additionally, circAXIN1, highly expressed in gastric cancer tissues and cell lines, encodes a 295aa protein, AXIN1-295aa [196]. The targeted silencing of circAXIN1 using siRNA significantly diminishes cancer-related traits such as cell growth, migration, invasion, and colony formation. In stark contrast, exogenous expression of AXIN1-295aa accelerates cancer advancement. The oncogenic role of AXIN1-295aa stems from its interaction with APC, a component crucial for the degradation of β-catenin. This interaction leads to a decrease in the binding between AXIN1 and APC, consequently elevating β-catenin activity, which further contributes to the progression of gastric cancer. These findings underscore the potential of targeting the Wnt/β-catenin signaling pathway as a treatment strategy for this type of cancer.

MET, a key receptor tyrosine kinase for GBM, is activated by the hepatocyte growth factor (HGF). A recent study found that circMET produces a MET variant, MET404, via m^6^A-modification, driven specifically by YTHDF2 instead of YTHDF1/3, which are known m^6^A readers for m^6^A-mediated translation [156]. MET404 is often found to be overexpressed in GBM cases. It acts as a secreted protein that binds to the MET β subunit, leading to constitutive activation of the MET receptor in GBM, a significant factor in GBM tumorigenesis and associated with a poor prognosis. Targeting MET404 with a neutralizing antibody, or using the FDA-approved MET inhibitor, onartuzumab, either alone or in combination, results in decreased tumor growth and extended survival in vivo. This suggests a potential treatment approach for GBM.

A comprehensive understanding of complex signaling networks and their dysregulation in various cancers is essential for developing safer and more effective therapeutic approaches. As research advances, the potential to harness circRNAs and their translated products for targeted cancer interventions is continually expanding, paving the way for improved cancer treatments.

## Conclusion and future directions

In recent years, a surge of research has illuminated various non-canonical translation mechanisms, diverging from the classical paradigm dependent on a cap, AUG start codon, stop codon, and poly(A)-tail. Cancer cells employ various non-canonical translation mechanisms to ensure survival, maintain stemness, and adapt to different environments, including the development of drug resistance.

This review highlights recent progress in non-canonical translation mechanisms, underscoring their emerging therapeutic potential in oncology. The exploration of these mechanisms, including non-canonical ORFs, m^6^A-modification, and circRNAs, holds the promise of unveiling uncharted territories in the human proteome. Novel isoforms and proteins arising from these unconventional processes may have dual roles in tumorigenesis. Targeting the translation machinery or modulating signaling pathways governed by tumor-promoting peptides/proteins shows promise for cancer therapy. For peptides/proteins that suppress tumors, activating the critical downstream pathway or delivering the peptide or circRNA could be a viable target for drug development. However, our comprehension of these mechanisms in cancer is progressing, necessitating further in-depth research.

Moreover, an imperative task lies in delineating the precise roles and implicated signaling pathways of these novel peptides/proteins across diverse biological contexts within the cancer milieu. Despite these challenges, conceptualizing and implementing a diverse array of strategies to impede non-canonical translation pathways offers profound potential as a therapeutic avenue in the expansive landscape of cancer treatment.

## Data Availability

All data generated or analyzed during this study are included in this published article.
